# What does Chinese BERT learn about syntactic knowledge?

**DOI:** 10.7717/peerj-cs.1478

**Published:** 2023-07-26

**Authors:** Jianyu Zheng, Ying Liu

**Affiliations:** Department of Chinese Language and Literature, Tsinghua University, Haidian Distinct, Beijing, China

**Keywords:** Chinese, BERT, Syntax, Fine-tune, NLP

## Abstract

Pre-trained language models such as Bidirectional Encoder Representations from Transformers (BERT) have been applied to a wide range of natural language processing (NLP) tasks and obtained significantly positive results. A growing body of research has investigated the reason why BERT is so efficient and what language knowledge BERT is able to learn. However, most of these works focused almost exclusively on English. Few studies have explored the language information, particularly syntactic information, that BERT has learned in Chinese, which is written as sequences of characters. In this study, we adopted some probing methods for identifying syntactic knowledge stored in the attention heads and hidden states of Chinese BERT. The results suggest that some individual heads and combination of heads do well in encoding corresponding and overall syntactic relations, respectively. The hidden representation of each layer also contained syntactic information to different degrees. We also analyzed the fine-tuned models of Chinese BERT for different tasks, covering all levels. Our results suggest that these fine-turned models reflect changes in conserving language structure. These findings help explain why Chinese BERT can show such large improvements across many language-processing tasks.

## Introduction

Bidirectional Encoder Representations from Transformers (BERT) (Devlin et al., 2019), a type of pre-trained language model, has been widely used in the natural language processing (NLP) community (Peng, Yan & Lu, 2019; Choi et al., 2020). BERT has greatly improved the effects of many NLP tasks (Wang et al., 2018). Therefore, researchers have started to explore the cause of BERT’s excellent performance (Rogers, Kovaleva & Rumshisky, 2020) and what knowledge BERT learned from the corpus during pre-training (Wu et al., 2020; Tenney, Das & Pavlick, 2019). In other words, there has been a focus on the interpretability of the model (Ranaldi, Fallucchi & Zanzotto, 2022). Most of the work in this area has centered on the knowledge, such as lexicon (Ravichander et al., 2020), syntax (Htut et al., 2019; Clark et al., 2019), and reasoning competence (Aken et al., 2019) learned by English BERT.

Unlike English, Chinese sentences involve a sequence of characters without explicit word boundaries (Wang, Cui & Zhang, 2020). Relatively little research has been conducted on the interpretability of Chinese BERT (Wang, Cui & Zhang, 2020; Koto, Lau & Baldwin, 2021; Xiang et al., 2021). Chinese BERT stores the information about the relationships between characters, and previous works have studied the word structure captured by Chinese BERT (Wang, Cui & Zhang, 2020). However, no research has ever explored whether Chinese BERT has determined the relationship between words composed of characters, as well as the syntactic information by which words can be organized into sentences. The research on the syntactic knowledge encoded in Chinese BERT can not only reveal the reasons why the model has achieved superb performance in many NLP tasks, but also guide the design of a more targeted model. Therefore, this work aimed to explore the syntactic ability of this model, Chinese BERT.

**Figure 1:**
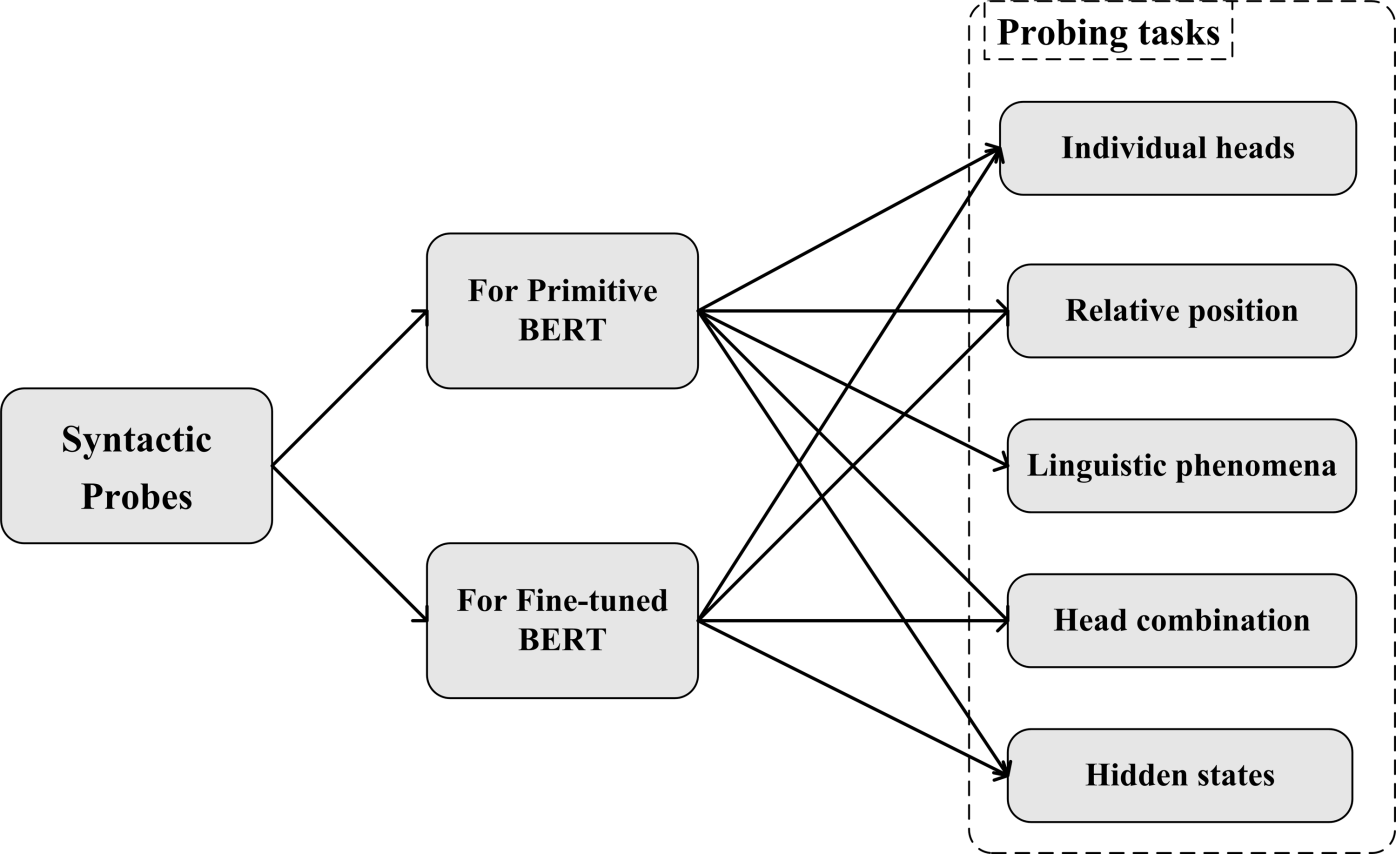
Illustration of syntactic probes.

We designed a series of probing experiments, shown in Fig. 1. Our probs can be classified into two parts: for original BERT and for fine-tuned BERT. For probing the original Chinese BERT, each attention head of Chinese BERT was firstly detected. When a sentence was input, the attention information between words was represented by each head of each layer, which is the attention matrix of the sentence. We tested whether a specific head existed so that a certain type of dependency relationship could be better determined and exceed the baseline. We then explored whether the attention head was sensitive to the relative position in syntactic relations. According to the particular linguistic phenomena in Chinese, we investigated Chinese BERT’s ability in some typical sentence structures, such as “bèi” construction, “baˇ” construction, and sentences using particles “zhe”, “le”, and “guò” to express aspects. Next, we combined all heads in the model to detect the prediction performance on the entire syntactic relationship. Additionally, we studied the syntactic knowledge learned by the hidden state of each layer. Following Conneau et al. (2018), we designed three syntactic tasks in the Chinese version and developed the corresponding datasets, namely tree depth, bigram shift, and dependency relation. By adding a simple classifier on the hidden state, we explored whether syntactic knowledge was learned by hidden representations, according to the results of the classifier on the three syntactic tasks. For probing fine-tuned Chinese BERTs, we fine-tuned Chinese BERT to downstream tasks at different levels. By comparing our results with the original Chinese BERT, we explored whether there were changes in the syntactic knowledge stored in the fine-tuned models.

Our experiments showed that no individual attention head could effectively learn the overall syntactic relationship, but some heads did capture the corresponding relationships. By combining attention heads, BERT could parse a sentence well, meaning that BERT’s attention heads encoded a large amount of syntactic knowledge. In addition, some attention heads were able to learn certain linguistic phenomena in Chinese. Through probing relative positions, we found that the performance of heads became worse as the distance between the dependent word and head word increased. As for hidden states, syntactic information was embedded in each layer to various degrees. When fine-tuning into downstream tasks, we observed the changes in conserving syntactic knowledge. Part-of-speech (POS) tagging strengthened syntactic information in Chinese BERT to some extent, while natural language inference (NLI) enabled Chinese BERT to forget plenty of knowledge in the language’s structure.

To our best knowledge, we are the first to investigate syntactic knowledge in Chinese BERT from different perspectives, including attention heads, hidden states, and downstream tasks. In addition, although our research took the most representative Chinese language model, Chinese BERT, as research object, our approaches and thoughts could be generalized to study other Chinese language models.

Our contribution can be summarized as the following:

(1) By referring previous work, we made out a series of comprehensive probes on attention heads about Chinese syntactic knowledge. Then we provided detailed analysis of these probing results.

(2) We modified the previous probing measure, which could be more applicable to Chinese with a character-based sequence.

(3) We evaluated linguistic phenomena learned by attention heads, and tested the impact of relative position on capturing syntactic knowledge.

(4) We released the Chinese datasets about three syntactic tasks: Bigram Shift (BShift), Tree Depth (TreeDepth), and Dependency Relation (DepRel).

## Related Work

Researchers have proposed many methods to investigate the syntactic knowledge that English BERT has learned. Clark et al. (2019) probed each attention head for various syntactic relationships by calculating accuracy in terms of the attention weights of the most-attended-to other word of each input word, and then they combined all attention heads to measure the overall dependency parsing ability. Hewitt & Manning (2019) used a structural probe to investigate whether syntax trees were embedded into a word representation space of the neural network by way of linear transformation. They concluded that the syntactic trees could be relatively recovered. In addition to exploring attention heads, some researchers have studied syntactic knowledge stored in hidden states. Tenney et al., (2019) designed a classifier on the span representations to probe syntactic knowledge in BERT. They concluded that BERT encodes syntax more than semantics. Goldberg (2019) fed complete sentences into BERT while masking out the single focus verb and then asked BERT for word predictions of the masked position. It was determined that BERT learns significant knowledge of syntax, particularly subject-verb agreement. Dai, Kamps & Sharoff (2022) used some syntactic probing tasks to analyze the performance of BERT’s syntactic dependencies and demonstrated that BERT “knows” about these knowledge. In addition, they also found that BERT’s ability to recognize syntactic dependencies often decreases after fine-tuning for NMT tasks. Besides, Ranaldi & Pucci (20230) found that syntactic knowledge could be acted as a point to test the connection between the empirism in real world and the knowledge derived from BERT. Based on the probing works in English, Ningyu et al. (2022) evaluated the cross-lingual syntactic relations in mBERT. They overlaid a linear classifier to decode the syntactic relation between head word and dependent word of each language.Then visualized the output representations of each classifier to analyze and summarize relations among languages. The above research was an insightful reference for our study.

Another line of work has studied the linguistic knowledge that Chinese BERT has encoded. Wang, Cui & Zhang (2020) investigated word features in Chinese BERT according to attention weight and some probing tasks, including Chinese Word Segmentation (CWS) and various-level downstream tasks in NLP. They found that some attention heads can implicitly capture word structure, and different Chinese tasks rely on word information to different degrees. Koto, Lau & Baldwin (2021) introduced seven discourse-related probing tasks to explore the discourse structure that Chinese BERT has learned. By adding an MLP layer on top of the model, they tested the accuracy of the classifier on predicting the competence of Chinese BERT comprehending discourse structure. Xiang et al. (2021) constructed the corpus of Chinese linguistic minimal pairs (CLiMP) to study the knowledge that Chinese language models have acquired, including 16 grammatical contrasts in Mandarin, covering nine major Mandarin linguistic phenomena. However, their work did not explore what syntactic relationship Chinese language models have learned. They still determined the competence of models’ language understanding in terms of the accuracy of the classifier on representation. Based on those works, we explored the syntactic knowledge of Chinese BERT across various aspects, including attention heads, hidden-state representation, and downstream tasks. The experimental results also showed Chinese BERT’s abilities more thoroughly.

## Background: Chinese BERT

We chose Chinese BERT, a very representative transformer-based model (Vaswani et al., 2017), as the target for analysis. Chinese BERT (Devlin et al., 2019) is pre-trained on Chinese simplified and traditional text from a Chinese Wikipedia dump of about 0.4 billion tokens.

In this work, we used the PyTorch implementation of Chinese BERT. All our experiments were based on the BERT-based-Chinese model. This model contained 12 layers, and each layer had 12 attention heads (110M parameters). Given a Chinese sentence *s* = *c*_1_, *c*_2_, …, *c*_n_, *c*_i_ delegated a token in the sentence. An attention head took as input vectors a sequence of *e* = [*e*_1_, *e*_2_, …, *e*_n_], which corresponded to n tokens. For each token vector ei, an attention head transformed it into query (*q*_i_), key (*k*_i_), and value (*v*_i_) vectors. An output vector (*h*_i_) could be obtained *via* a weighted sum of value vectors based on attention distribution (*α*), a kind of weight matrix between all pairs of tokens. Attention distribution can be calculated using the dot product with a softmax function between the query and key vectors.

\begin{eqnarray*}{\alpha }_{ij}= \frac{\exp \nolimits ({q}_{i}^{T}{k}_{j})}{\sum _{l=1}^{n}\exp \nolimits ({q}_{i}^{T}{k}_{l})} \end{eqnarray*}

\begin{eqnarray*}{h}_{i}=\sum _{j=1}^{n}{\alpha }_{ij}{v}_{j}. \end{eqnarray*}

The output vector *h*_i_ represents the hidden state of a head about token *c*_i_. The hidden states of all heads from the same layer can be concatenated to obtain a hidden representation ${\hat {\mathrm{h}}}_{\mathrm{i}}$about token *c*_i_.

\begin{eqnarray*}{\hat {\mathrm{h}}}_{\mathrm{i}}=[{h}_{i}^{1}, {h}_{i}^{2},\ldots ,{h}_{i}^{n}] \end{eqnarray*}

where ${h}_{i}^{\mathrm{j}}$ represents the hidden state of *j*-th head ot token *i*.

When preprocessing the input text, the special tokens [CLS] and [SEP] were added to the beginning and end of each sentence, respectively. Chinese BERT is pretrained on two tasks: masked language modeling (MLM) and next sentence prediction (NSP). The MLM task predicts the words masked randomly in the input, while NSP determines whether a sentence is subsequent to another in the original document.

## Probing Tasks

It has been reported that BERT can implicitly encode linguistic knowledge (Jawahar, Sagot & Seddah, 2019). To identify what knowledge Chinese BERT has learned, some experiments have been designed to probe it. In this work, we first adopted two Chinese Dependency Treebanks as golden datasets for experiments and evaluation. Then we designed two types of probing tasks: attention-based tasks and hidden-state-based tasks. Attention-based tasks include probing individual attention heads, relative positions, and linguistic phenomena in Chinese which the attention head has learned. Hidden-state-based tasks evaluate the syntactic competence stored in the hidden state according to three syntactic tasks.

### Datasets

Different treebanks exist, with divergence in their annotation guidelines and corpus sources. We chose two representative Chinese dependency treebanks for our experiments: the Chinese Universal Dependencies treebank 2.11(UD 2.11) (https://universaldependencies.org/) and Chinese Dependency Treebank 1.0 (CDT 1.0) (https://catalog.ldc.upenn.edu/LDC2012T05).

Universal Dependency is an open community covering nearly 200 treebanks in over 100 languages. We selected all Chinese treebanks from Universal Dependencies 2.11. The Chinese Universal Dependencies treebanks contain 8,460 sentences (161,856 words). The annotation guidelines can be found in Marneffe et al. (2021). Chinese Dependency Treebank 1.0 was released by the Harbin Institute of Technology Research Center for Social Computing and Information Retrieval (HIT-SCIR). From the People’s Daily newswire stories published between 1992 and 1996, 49,996 Chinese sentences (902,191 words) were randomly selected. For more details about the annotation guidelines, please refer to Che, Li & Liu (2012). We shuffled the data for subsequent experiments.

### Probing individual attention heads

#### Setup

In this subsection, we probed which individual heads could best learn dependency relations. When we input a sentence into Chinese BERT, we obtained the attention matrix about characters in this sentence for each head. Considering that no explicit word boundary exists in Chinese sentences, we used the word segment of datasets in ‘Datasets’ as the standard. Then, we summed the columns and averaged the rows corresponding to the constituent characters of the standard words:

\begin{eqnarray*}{\alpha }_{{w}_{p}\rightarrow {w}_{q}}= \frac{1}{{|}{w}_{p}{|}} \sum _{{\mathrm{c}}_{\mathrm{i}}\in {\mathrm{w}}_{\mathrm{p}}}\sum _{{\mathrm{c}}_{\mathrm{j}}\in {\mathrm{w}}_{\mathrm{q}}}{\alpha }_{{c}_{i}\rightarrow {c}_{j}} \end{eqnarray*}

where, *w*_p_ and *w*_q_ are the words in the input sentence. *c*_i_ and *c*_j_ are the constituent characters in words *w*_p_ and *w*_q_, respectively. *α* ∈(0, 1) ^n×n^ is the attention weight of a certain head regarding the input sentence. — *w*_p_— is the number of characters in *w*_p_.

**Figure 2:**
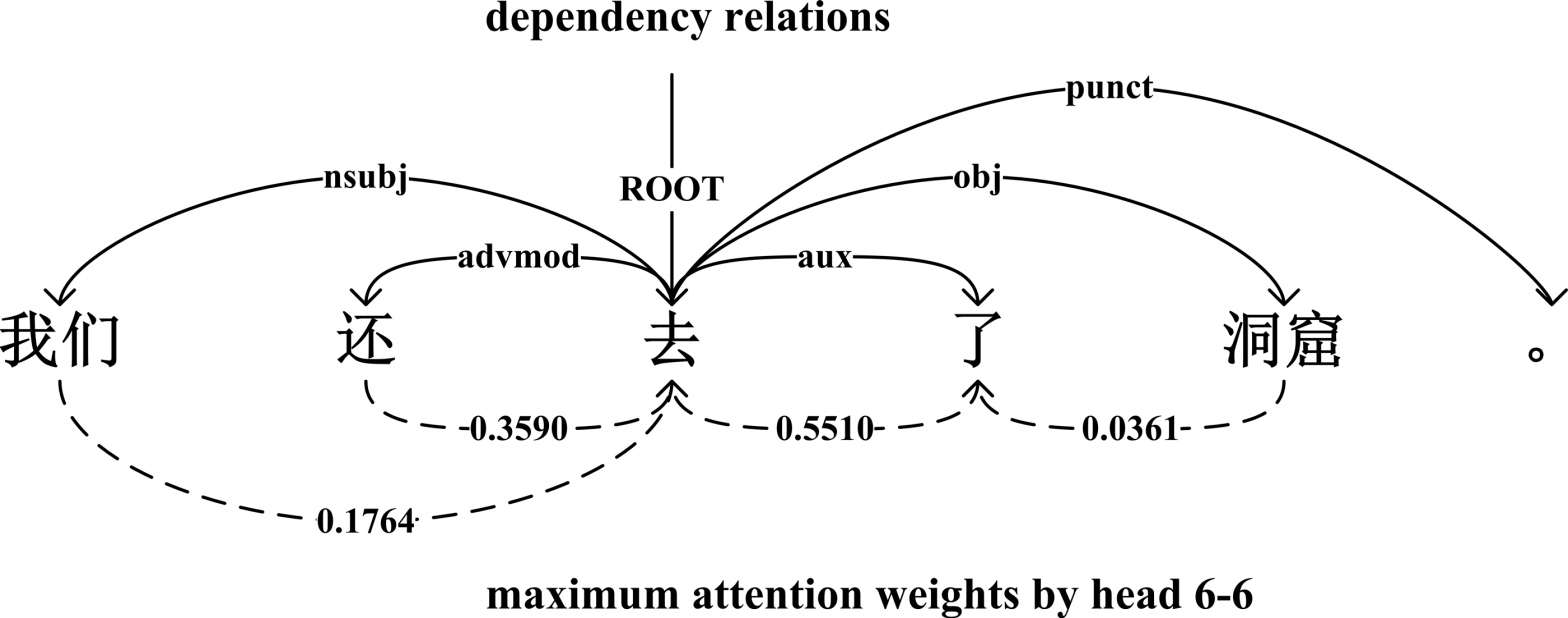
An example sentence parsed by dependency relations and maximum attention weights.

Figure 2 shows that an example sentence parsed by dependency relations and expressed by attention weights from head 6-6. If an attention head learned a certain dependency relation well, this head had a higher probability of allocating the maximum weight to the head word in each row of the attention matrix. During the evaluation, we ignored the direction between the dependent word and head word, and tested the performance of each attention head on each dependency relation and overall relations. We used the undirected unlabeled attachment score (UUAS) as our evaluation:

\begin{eqnarray*}UUAS= \frac{\mathrm{c}orrec{t}_{\mathrm{i}}^{\mathrm{k}}}{{|}{\mathrm{rel}}_{i}{|}} \end{eqnarray*}

where, —*rel*_i_— is the number of dependency relation *i* in the datasets, and ${\text{correct}}_{i}^{\mathrm{k}}$ is the number of correct predictions of relation *i* for a given head *k*.

#### Baselines

We adopted positional offset and Random BERT as baselines. For the positional offset baseline, we determined the most common position where the head word could occur for each attention word. For the Random BERT baseline, we used a BERT-base model with randomly initialized weights.

#### Results

Tables 1 and 2 show the results of our probing method and baselines on UD2.11 and CDT1.0, respectively. The number in the parentheses in the line “positional offset” is the offset location with the best performance (*e.g.*, (-1) means the head word was located to the left of the dependent word). The number in the parentheses in the line “Chinese BERT” denotes the best performance head, *i*-*j* denotes the *j*-th head in the *i*-th layer. The 17 most common relations are shown in Table 1 and all relations are shown in Table 2.

**Table 1:** UUAS on UD2.11. The values with the bold style are the maximum values in each column among these methods or models.

**Model**	**Total**	**nmod**	**nsubj**	**obj**	**case**	**compound**	**nummod**	**advmod**	**mark**
Positional Offset	27.1(1)	40.7(1)	32.2(1)	28.4 (−1)	34.8 (−1)	90.3(1)	91.8(1)	57.6(1)	33.4 (−1)
Random BERT	6.2	7.0	7.9	5.8	8.1	6.7	11.1	8.8	5.8
Chinese BERT	**35.1** (5–5)	**61.3** (7–11)	**44.4** (7–11)	**74.6** (7–2)	**37.8** (4–10)	**90.3** (6–3)	**92.3** (7–4)	**61.7** (5–5)	**51.8** (6-11)
**Model**	**advcl**	**conj**	**obl**	**aux**	**ccomp**	**clf**	**amod**	**parataxi**	**xcomp**
Positional Offset	12.8(2)	28.0 (−2)	34.8(1)	47.5 (−1)	14.1 (−3)	34.1(1)	43.8(1)	3.4 (−3)	38.3 (−1)
Random BERT	6.4	8.4	7.9	7.1	5.4	9.1	8.7	10.4	6.5
Chinese BERT	**31.3** (8–7)	**58.2** (8–11)	**41.0** (5–9)	**74.7** (5–5)	**42.7** (5–7)	**72.1** (8–8)	**68.6** (8–5)	**17.0** (11–1)	**46.1** (7–2)

**Table 2:** UUAS on CDT1.0. The values with the bold style are the maximum values in each column among these methods or models.

**Model**	**Total**	**ATT**	**ADV**	**VOB**	**SBV**	**COO**	**RAD**
positional offset	27.9(1)	61.6(1)	48.5(1)	28.1 (−1)	44.0(1)	18.7 (−2)	**74.7** (−1)
random BERT	6.1	8.2	6.1	6.3	7.3	7.3	8.9
Chinese BERT	**35.1** (9-9)	**65.2** (9–9)	**54.8** (6–6)	**65.8** (6–8)	**52.6** (9–3)	**44.5** (6–1)	**74.7** (7–4)
**Model**	**POB**	**CMP**	**LAD**	**FOB**	**DBL**	**IOB**	
positional offset	34.2 (−1)	73.8 (−1)	58.3(1)	48.7(1)	50.0 (−1)	78.2 (−1)	
random BERT	9.2	7.6	9.7	7.7	8.2	10.6	
Chinese BERT	**77.7** (8-3)	**83.5** (7–8)	**73.0** (9–6)	**68.3** (9–10)	**69.5** (8–3)	**85.9** (8–3)	

From the two tables, we found that Chinese BERT >Positional Offset >Random BERT in terms of performance. This indicated that the attention heads in Chinese BERT learned some dependency relations implicitly, while Random BERT captured very little syntactic knowledge (<10%). Meanwhile, positional offset performed similarly on some dependency relations, such as “compound” in Table 1 and “RAD” in Table 2. This could be because the head word appeared fixed in the distance of the dependent word. The attention head could only learn positional or distance information between the two words to achieve general performance.

In addition, we also found that some dependency heads did significantly learn some specific syntactic relations, sometimes achieving high accuracy, such as “obj” and “aux” in Table 1, and “VOB” and “POB” in Table 2. However, no single heads performed well on the total relations. The best single heads only obtained 35.1 UUAS on the two datasets. This finding is similar to the work of Clark et al. (2019) on English treebanks. We also found that most of the heads with the best performance in the specific dependency relations were located in the middle layers (layers 5–9). This was due to the fact that Chinese BERT encodes how to organize words into a sentence mostly in the middle layer, similar to English BERT (Jawahar, Sagot & Seddah, 2019).

### Probing relative position

#### Setup

According to the previous subsection, we found that positional offset could also achieve good performance on some dependency relations. Therefore, we investigated whether the distance between dependent words and head words could affect the performance of Chinese BERT in capturing syntactic knowledge. UUAS was still used as our evaluation metric for this experiment.

#### Baselines

We adopted Random BERT as a baseline. For the full details, please refer to ‘Probing individual attention heads’.

#### Results

**Figure 3:**
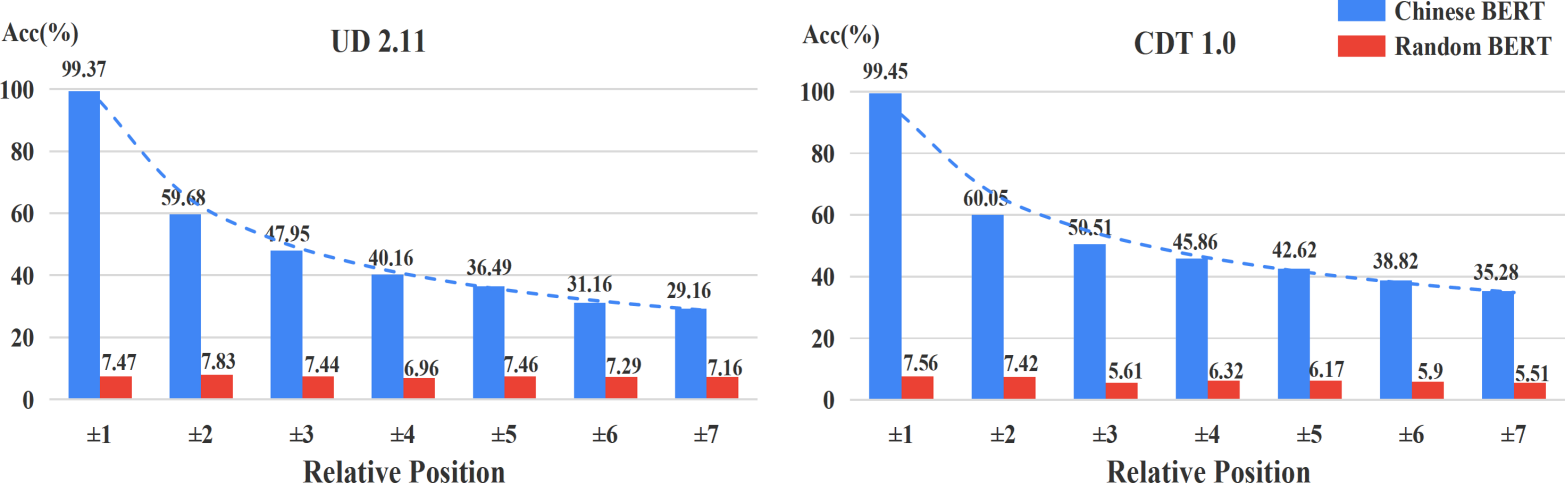
Accuracy of relative positions.

Figure 3 shows the accuracy of relative positions on UD2.11 and CDT1.0, respectively. We also found that Chinese BERT apparently exceeds Random BERT in different positional distributions. In addition, the performance of Chinese BERT decreased as the distance increased. This indicates that positional information between words is important for Chinese BERT. The closer the distance between the head word and dependent word, the better Chinese BERT can capture the dependency relation between the two. Among all relative positions, Chinese BERT achieves very high performance (>99%) when the head word and dependent word are next to each other (±1).

**Figure 4:**
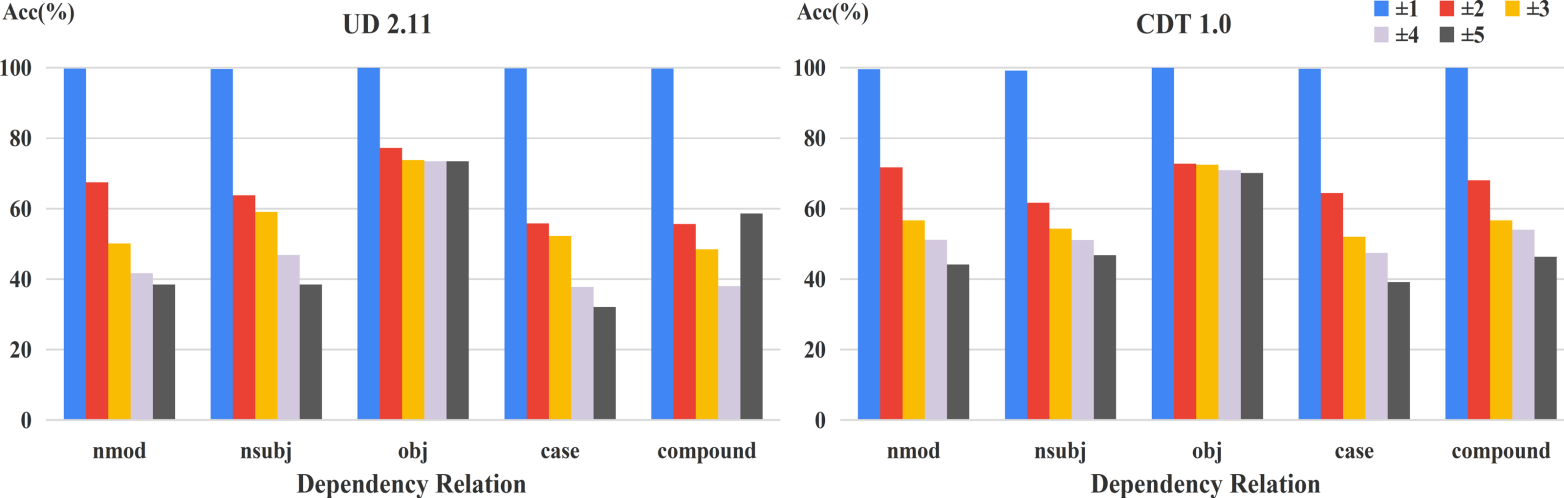
Accuracy of relative positions on common relations.

Furthermore, in order to analyze the influence of positional distribution on different dependency relations, we calculated the accuracy of relative positions on the common relations of the two datasets, shown in Fig. 4. From this figure, we can easily see that the relation between model performance and dependency distance still exists in most dependency relations.

### Probing linguistic phenomena in Chinese

#### Setup

Based on the findings from the previous subsections, we were very interested in exploring particular linguistic phenomena existing in Chinese, as well as determining whether Chinese BERT had captured them. Hence, we designed a test suite for evaluation.

Our test suite covered two sentence constructions unique in Chinese and three auxiliary words for expressing aspects in Chinese sentences. For sentence construction, we chose baˇ (把) construction and bèi (被) construction. For auxiliary words, -zhe (着), -le (了), and -guò (过) were adopted.

The particle baˇ (把) is commonly used in Chinese. It can change the word order from “subject - verb - object” to “subject - baˇ - object - verb” (Ye, Zhan & Zhou, 2007). The construction is always used to express the result of the action on the object. Different from English, the bèi (被) construction is used to express passive voice in Chinese. Due to lack of morphological inflection, the particle bèi as a fixed word is used before an agent to express passive voice (Wang & Xu, 2015). The basic structure consists of “object - bèi - subject - verb - other components”. The particles -zhe (着), -le (了), and -guò (过) in Chinese can come after a verb to express aspects in Chinese sentences. The durative aspect can be reflected by the marker -zhe, which describes an enduring or continuing situation. The perfective aspect can be expressed by the markers -le and - guò. The perfective particle -le expresses a situation in its entirety, an event bounded at the beginning and the end, while the other perfective particle -guò presents an event that has been experienced at some indefinite time (Chen & Shirai, 2010). We give some example sentences for illustration:

(1) 他把杯子打破了

(He ba cup break le.)

He broke the cup.

(2) 杯子被他打破了。

(Cup bei he break le.)

The cup was broken by him.

(3) 他去北京了。

(He go Beijing le.)

He went to Beijing.

(4) 他去过北京。

(He go guo Beijing.)

He has been to Beijing.

(5)门开着。

(Door open zhe.)

The door is open.

In our experiment, we probed the model’s competence at capturing these phenomena through an attention matrix. Specifically, we measured whether those particles allocated the maximum attention weight to their dependency heads.

#### Baselines

We adopted positional offset and Random BERT as baselines. For the full details, please refer to ‘Probing individual attention heads’.

#### Results

Our experimental results are displayed in Table 3. The number in the parentheses is the specific head with the best performance. The performance still illustrates: Chinese BERT >Positional Offset >Random BERT. Meanwhile, we also saw that the results of Chinese BERT and Positional Offset were the same on the particles -zhe (着) and -guò (过). By analyzing the corpus, we found that -zhe and -guò followed the main verb most of time, indicating that Chinese BERT could only learn some positional information used in predicting the dependency relations of the two particles. In particular, we discovered that the two constructions (bèi and baˇ) were learned very well by the heads in the middle layers (layers 4–6), while the three particles (-zhe, -le, and - guò) were captured best by the heads in the lower layers (layers 2–4). This indicates that the structure information about sentences exists in the middle layers. Some lexical or morphological knowledge is embedded in the lower layers (Jawahar, Sagot & Seddah, 2019).

**Table 3:** Accuracy on Chinese linguistic phenomena. The values with the bold style are the maximum values in each column among these methods or models.

**Datasets**	**Model**	**baˇ(“把”)**	**bèi(“被”)**	**-zhe(“着”)**	**-le(“了”)**	**-guo(“过”)**
UD	random BERT	11.65	8.06	6.45	7.62	10.00
	positional offset	38.83	71.79	**100.00**	89.24	**100.00**
	Chinses BERT	**56.31** (6–10)	**87.52** (6–11)	**100.00** (2–1)	**89.97** (4–8)	**100.00** (2–1)
CDT	random BERT	6.51	7.22	5.05	5.55	4.38
	positional offset	31.64	59.18	**99.03**	90.40	**98.21**
	Chinses BERT	**84.66** (4–1)	**90.25** (6–11)	**99.03** (3–9)	**90.65** (4–8)	**98.21** (3–9)

### Probing attention head combinations

#### Setup

In ‘Probing individual attention heads’ we found that some single attention heads were good at learning the corresponding dependency relations, but no heads could capture the whole dependency structures of sentences. Hence, we considered to combine all heads to perform sentence parsing. We followed the setting from Clark et al. (2019) by training a classifier combing with all attention heads linearly:

\begin{eqnarray*}UUAS=soft\max \nolimits (\sum _{k=1}^{144}{w}_{k}{\alpha }_{ij}^{k}) \end{eqnarray*}

where softmax is a function for classification, 144 is the number of heads in Chinese BERT, *w*_k_ are weights for training, and ${a}_{ij}^{k}$ is the attention weight of word *i* on word *j* produced by head k. We refer to this method as “Attn”.

Additionally, we also considered the impact of words in carrying out parsing tasks. We incorporated word embeddings from Song et al. (2018) into the classifier. This method is called “Attn + embeddings”.

#### Baselines

Similar to Clark et al. (2019), “Random Initial Attention + embeddings”, “Right Branching”, and “Distances + Embeddings” were adopted as baselines in this experiment. “Random Initial Attention + embeddings” used a randomized network and incorporated the pre-trained word embeddings for head and dependent words. Meanwhile, “Right Branching” predicts that the head word was always on the right of the dependent. “Distances + Embeddings” is used to replace the attention matrix of Chinese BERT with pre-trained word and positional embeddings, and randomly initialized other weights.

#### Results

Results are exhibited in Table 4. We can see that both “Attn + embeddings” and “Attn” achieved better performances than the baselines on the two datasets. The accuracy of “Attn” was higher than 50%, and “Attn + embeddings” obtained nearly 70% accuracy. These results are similar to the findings in English (Clark et al., 2019; Hewitt & Manning, 2019). This indicates that the attention heads of Chinese BERT did acquire many organizational structures in language. “Attn + embeddings” outperformed “Attn”(∼15%), which proves that specific vocabulary contributes to Chinese BERT capturing dependency relations. Together with the findings from individual attention heads, we believe that Chinese BERT encodes abundant information in syntax by a way of indirect supervision, even though the word boundaries do not exist in the Chinese language.

**Table 4:** Accuracy on dependency parsing. The values with the bold style are the maximum values in each column among these methods or models.

**Methods**	**UD(%)**	**CDT(%)**
Random Init Attn + embeddings	11.47	11.01
Right Branching	29.59	31.37
Distances + embeddings	44.62	45.72
Attn	51.63	54.00
Attn + embeddings	**67.68**	**68.24**

### Probing hidden state

#### Setup

Besides probing attention heads, we explored the ability of hidden representation in capturing syntactic knowledge. Because no suitable datasets for testing Chinese syntax were available, we designed three Chinese syntactic tasks by imitating the work in English (Conneau et al., 2018): Bigram Shift (BShift), Tree Depth (TreeDepth), and Dependency Relation (DepRel).

In the BShift task, we inverted two random adjacent characters and let the model predict whether the sentence was inverted. In the TreeDepth task, the depth of the dependency tree of a sentence was predicted. The task DepRel refers to the prediction of the dependency relation of a phrase consisting of two words.

**Figure 5:**
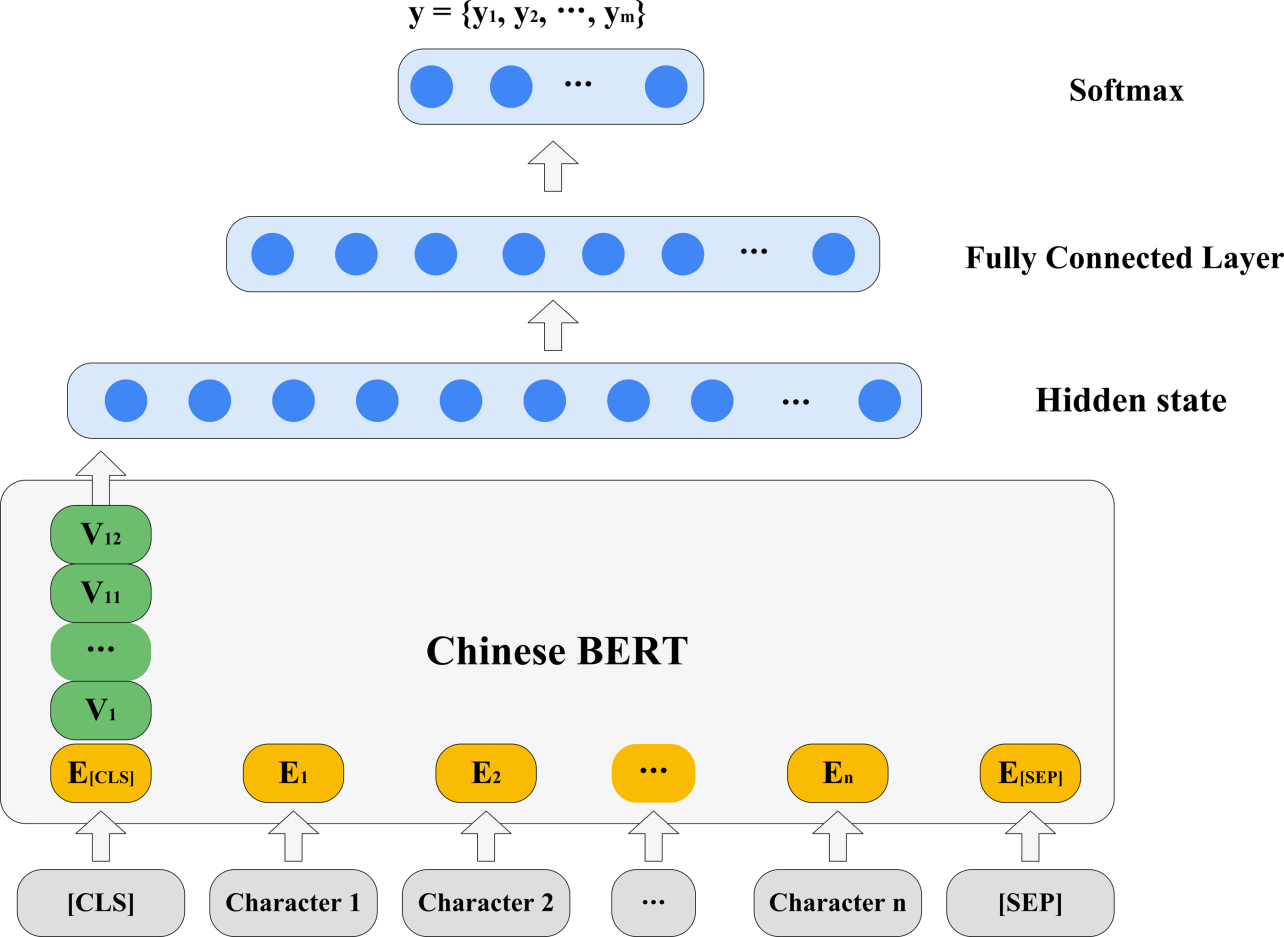
Probing syntactic knowledge in hidden states.

As shown in Fig. 5, we overlaid a one-layer MLP on the hidden state of each layer to construct a classifier. After trying different parameter combinations, an optimal set of parameters were finally determined. We then only trained each classifier one epoch, so that the classifier was forced to pay attention to information encoded in the hidden state representation as much as possible (Choenni & Shutova, 2020).

#### Baselines

We adopted Random BERT as a baseline. For the complete details, please refer to ‘Probing individual attention heads’.

#### Results

Table 5 displays the prediction result of each layer. The number in parentheses denotes the baseline from Random BERT. The bold numbers are the maximum values among all 12 layers in each task. According to the results, the best performances of Chinese BERT were all achieved in the final layer (layer 12). Chinese BERT still outperformed Random BERT in most of the layers across the three tasks. Compared to probing in the attention head, the performance of the hidden state from Random BERT was not very poor. This indicates that these hidden states contain some information that will contribute to predicting syntactic knowledge. Interestingly, we found the prediction results from Random BERT were better than Chinese BERT’s corresponding to lower layers (layers 1–3) in the TreeDepth task. This could be because a relatively complete structure is needed to be captured in the TreeDepth task. However, Chinese BERT may not encode structural information well in the lower layers. Therefore, its performance was outperformed by Random BERT.

**Table 5:** Accuracy on three syntactic tasks. The values with the bold style are the maximum values in each column among these methods or models.

**Layer**	**TreeDepth**	**BShift**	**DepRel**
**1**	42.32(43.44)	87.55(70.45)	84.71(76.76)
**2**	42.46(43.54)	86.30(71.03)	92.80(90.08)
**3**	40.99(43.77)	83.65(66.72)	89.26(93.80)
**4**	50.11(43.84)	91.78(75.59)	92.90(93.05)
**5**	51.28(45.31)	94.36(69.52)	94.43(94.28)
**6**	47.76(43.47)	87.50(68.43)	95.85(87.82)
**7**	44.00(42.85)	93.70(50.00)	95.72(75.44)
**8**	62.89(42.06)	76.41(57.76)	91.94(78.45)
**9**	75.13(43.78)	84.46(51.39)	93.73(75.69)
**10**	77.85(43.43)	80.97(50.93)	94.30(73.76)
**11**	78.03(43.08)	88.91(54.26)	95.70(73.67)
**12**	**79.82**(41.84)	**94.88**(50.13)	**97.87**(68.30)

## Fine-tuning on downstream tasks

When Chinese BERT is fine-tuned into downstream tasks, does its syntactic knowledge change? In order to explore this question, we selected tasks with different levels to fine-tune Chinese BERT. These tasks covered low-level tasks, such as word segment and POS tagging, and high-level tasks involving semantic comprehension, including NLI and question matching. We carried out the experiments on the following datasets:

**Word segment (WS)**. We adopted CTB8.0 (Xue et al., 2013) as the dataset.

**POS tagging**. CTB8.0 was used (Xue et al., 2013) as the dataset.

**NLI**. Original Chinese NLI (OCNLI)(Hu et al., 2020) is a CLUE task used to infer whether a premise sentence entails, contradicts, or is neutral towards a hypothesis sentence.

**Question matching (CQ)**. We used the Large-scale Chinese Question Matching Corpus (LCQMC) (Liu et al., 2019), which is a large-scale Chinese corpus.

We refer to these fine-tuned models as WS-BERT, POS-BERT, NLI-BERT, and CQ-BERT. These fine-tuned BERTs will be compared with the original Chinese BERT in the following experiments.We ran each downstream task three times and stored the model parameters. And our probing results are the averages of every three experiments.The findings will be described as follows.

### Probing individual attention heads for fine-tuned BERTs

**Figure 6:**
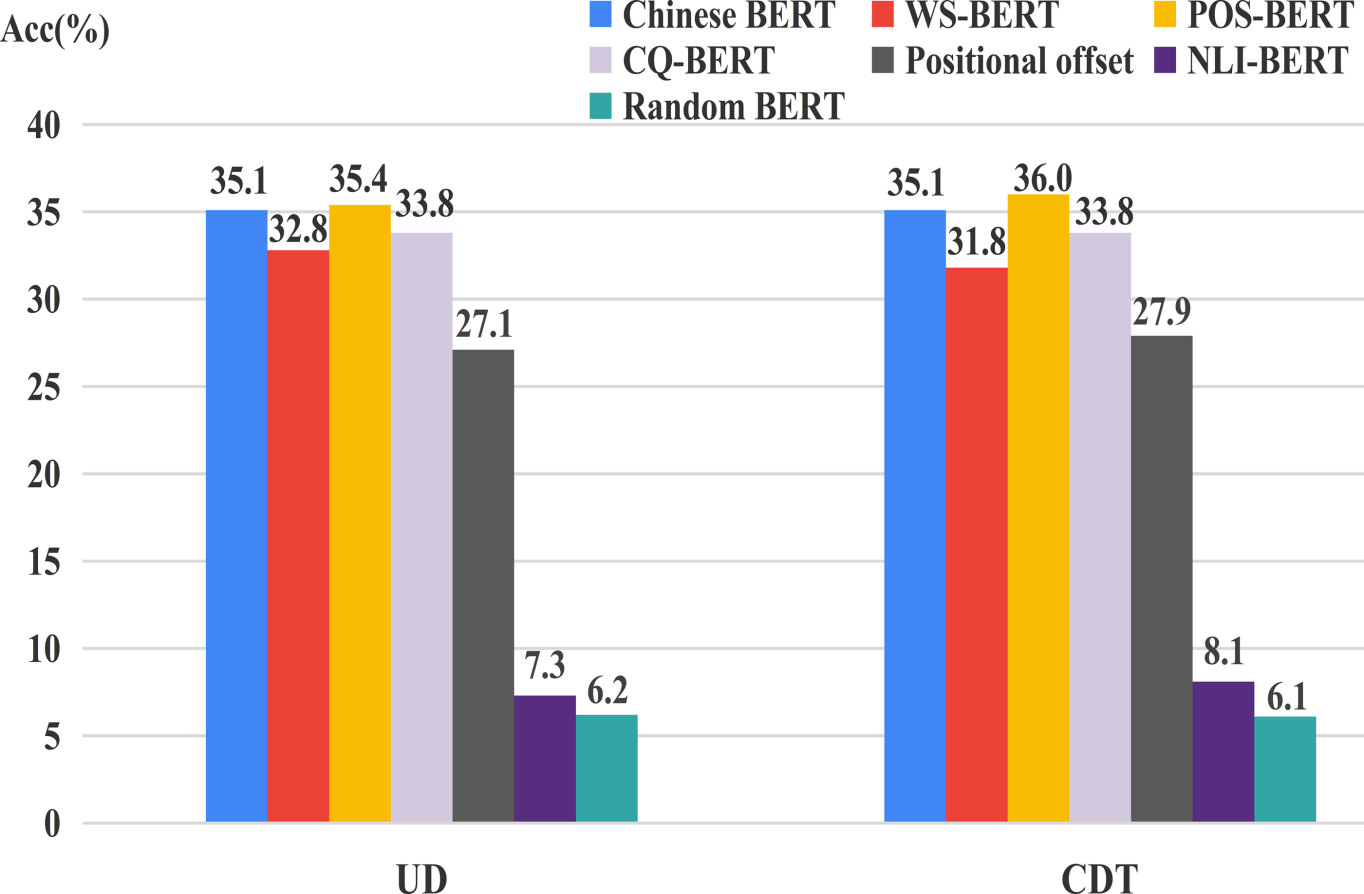
UUAS for fine-tuned models and Chinese BERT.

We still adopted positional offset and Random BERT as baselines. Figure 6 shows the UUAS of the individual heads on the overall relations for these different BERTs. One can easily see that the performance of NLI-BERT decreased dramatically (≈27%), suggesting that inference tasks do not need syntactic knowledge. Additionally, WS-BERT and CQ-BERT showed small loss consistently, which indicates that the two tasks could also forget some language structures during training. POS-BERT showed a little improvement compared to Chinese BERT. This may be because this task needed some relation information from surrounding words so that the POS of the current word could be identified more accurately.

**Figure 7:**
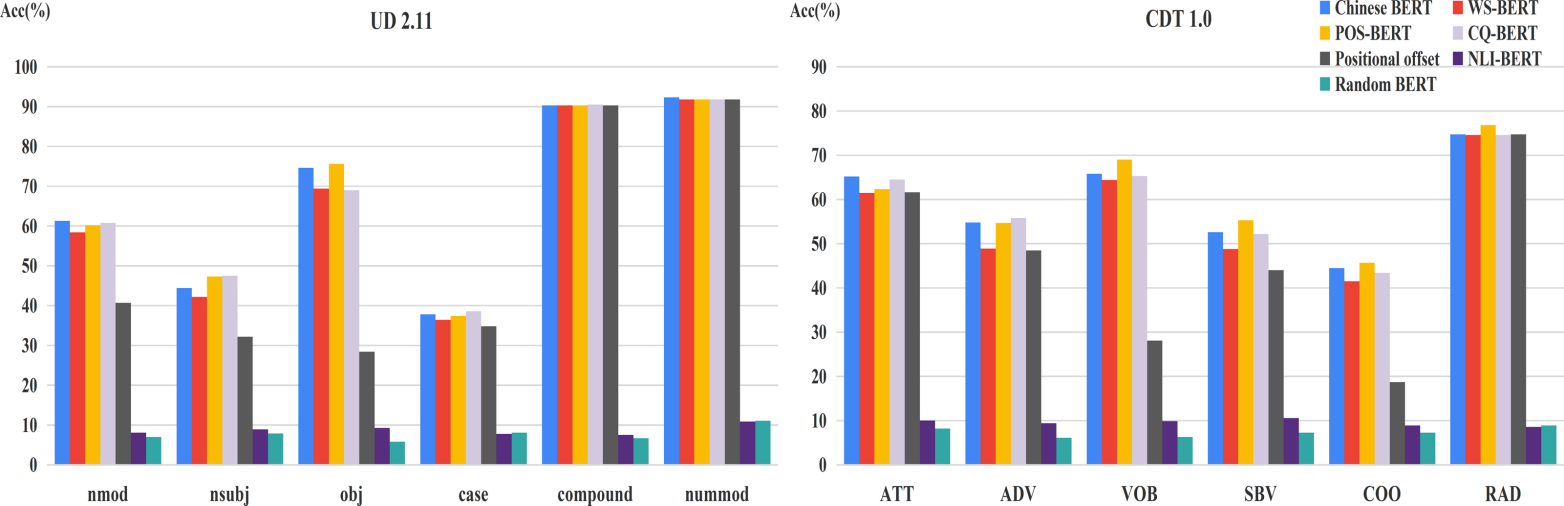
UUAS on common relations for fine-tuned models and Chinese BERT.

The accuracy results of the common relations of individual heads on fine-tuned BERTs are displayed in Fig. 7. Our findings from the overall relation are still roughly suitable to these frequent dependency relations. However, there exists some different cases. POS-BERT outperformed Chinese BERT on VOB and SBV. SBV and VOB act as the subject and object for a verb in a sentence, respectively (Marneffe et al., 2021). These relations could be useful for POS-BERT to determine the POS of a word. Also, Chinese BERT performed better than CQ-BERT on nmod, a kind of nominal modifier. This indicates that this relation could may not be necessary for the CQ task.

### Relative position for fine-tuned BERTs

The accuracy of relative positions for fine-tuned models and Chinese BERT is displayed in Table 6. We found that NLI-BERT only reserves some dependency knowledge in the relative position following fine-tuning. Compared with Chinese BERT, other BERTs maintain the performance when the dependent word and head word are next to each other. While the distance extends to two, these fine-tuned models improved their competence on capturing dependency relation. However, when the distance becomes longer, they are mostly exceeded by Chinese BERT. The reason could be that these fine-tuned BERTs pay more attention to local information between words. Therefore, when the dependent word and head word are very close, these fine-tuned models can obtain better results.

**Table 6:** Accuracy of relative positions for fine-tuned models and Chinese BERT. The values with the bold style are the maximum values in each column among these methods or models.

	**UD**						
**Methods**	**±1**	**±2**	**±3**	**±4**	**±5**	**±6**	**±7**
Chinses BERT	99.37	59.68	47.95	**40.16**	36.49	31.16	29.16
WS-BERT	99.58	**61.38**	47.32	36.10	34.38	29.63	26.65
POS-BERT	**99.60**	60.43	**49.01**	38.91	34.51	29.14	27.10
CQ-BERT	99.20	60.43	47.41	39.21	**36.56**	26.50	**29.48**
NLI-BERT	8.67	8.86	8.43	9.42	8.78	8.54	9.31

	**CDT**						
**Methods**	**±1**	**±2**	**±3**	**±4**	**±5**	**±6**	**±7**
Chinses BERT	99.45	60.05	50.51	45.86	**42.62**	**38.82**	35.28
CWS-BERT	99.64	60.05	48.58	42.90	40.29	35.06	31.32
POS-BERT	**99.69**	**62.17**	**52.10**	45.41	42.44	38.28	35.54
CQ-BERT	99.22	61.37	48.54	**46.12**	42.61	**38.82**	**35.66**
NLI-BERT	9.80	8.96	9.08	9.29	9.22	9.36	9.64

**Figure 8:**
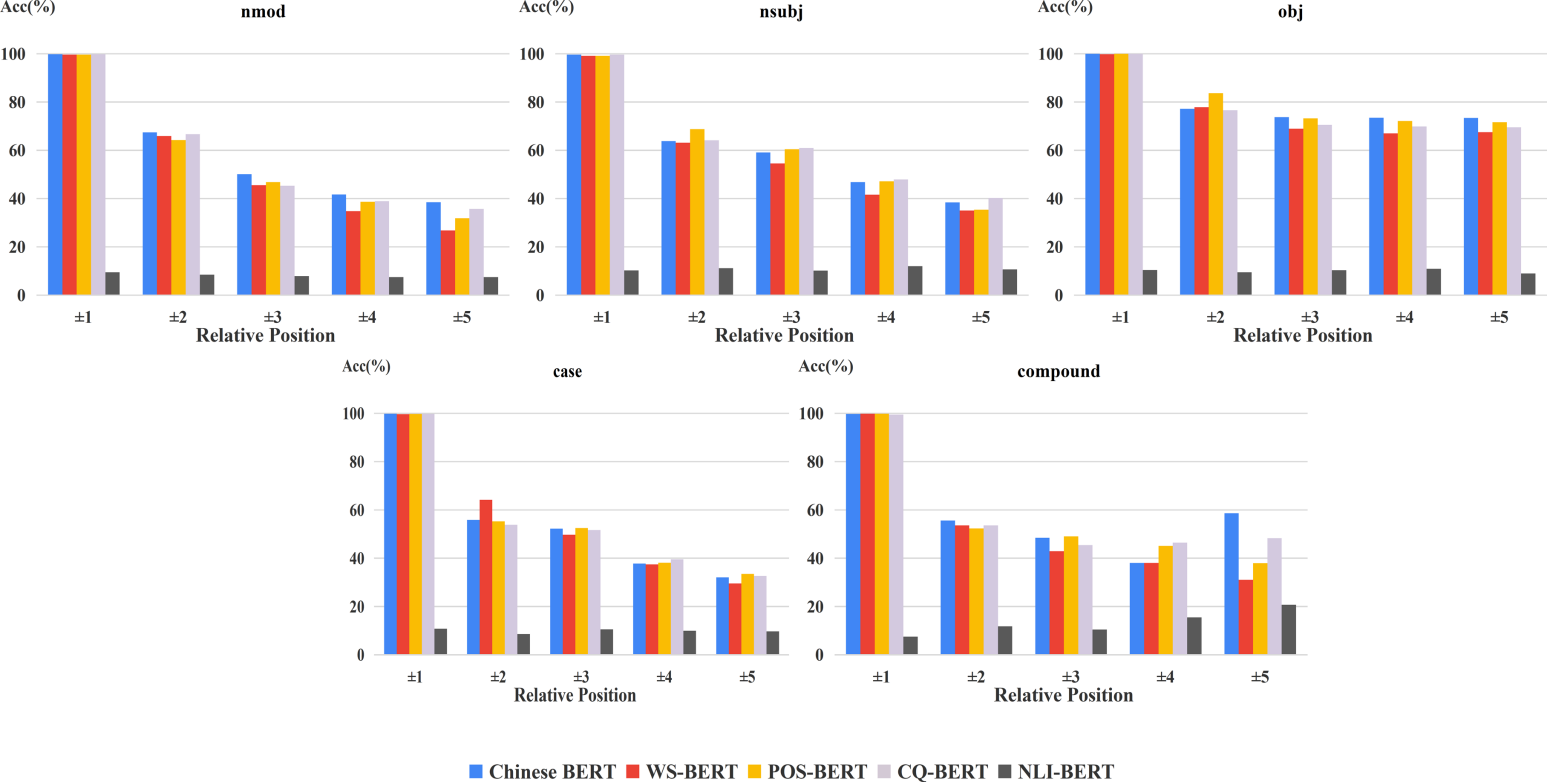
Accuracy of relative positions on UD2.11 for fine-tuned models and Chinese BERT.

**Figure 9:**
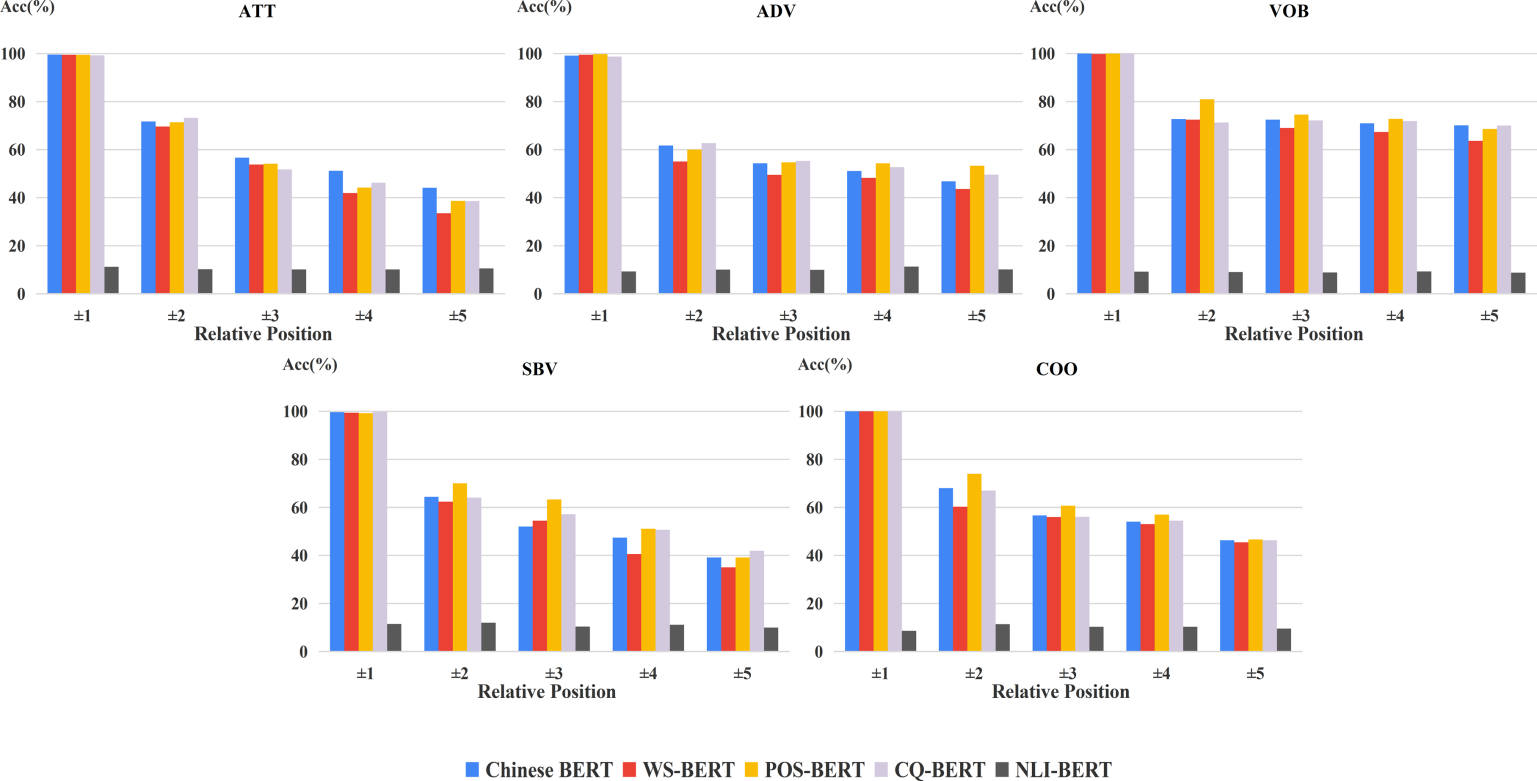
Accuracy of relative positions on CDT1.0 for fine-tuned models and Chinese BERT.

For exploring the changes of fine-tuned BERTs in frequent relations, we carried out the corresponding experiments on the two datasets (Figs. 8 and 9). In general, the margin between WS-BERT and Chinese BERT grew as the relative position became longer. This demonstrates that WS-BERT’s ability to preserve common syntactic relations decreases as the distance increases. Additionally, the performance gap between Chinese BERT and CQ-BERT in most relations remained small, indicating that dependency knowledge is not forgotten by CQ-BERT. POS-BERT’s performance surpassed Chinese BERT on some relations, such as nsubj, obj, VOB, SBV, and COO, which suggests that these relations are important for POS tagging task.

### Probing attention head combinations for fine-tuned BERTs

Table 7 shows the results of the dependency parsing accuracy after combining attention heads. The differences in performance among these BERTs were similar to the results in the previous subsections. Notably, POS-BERT outperformed Chinese BERT on CDT, but displayed a loss in performance on UD. We believe that this phenomenon is related to the smaller size of the UD dataset, so that the classifier failed to learn the information encoded in the fine-tuned BERTs.

**Table 7:** Accuracy on dependency parsing for fine-tuned models and Chinese BERT. The values with the bold style are the maximum values in each column among these methods or models.

**Methods**	**Chinese BERT**	**WS-BERT**	**POS-BERT**	**CQ-BERT**	**NLI-BERT**
	**UD**				
Attn	51.63	41.14	44.48	43.24	7.02
Attn + embeddings	**67.68**	**51.35**	**53.98**	**53.09**	**10.88**
	**CDT**				
Attn	54.00	49.93	56.62	53.20	7.07
Attn + embeddings	**68.24**	**64.21**	**69.59**	**66.80**	**10.17**

### Probing hidden state for fine-tuned BERTs

**Figure 10:**
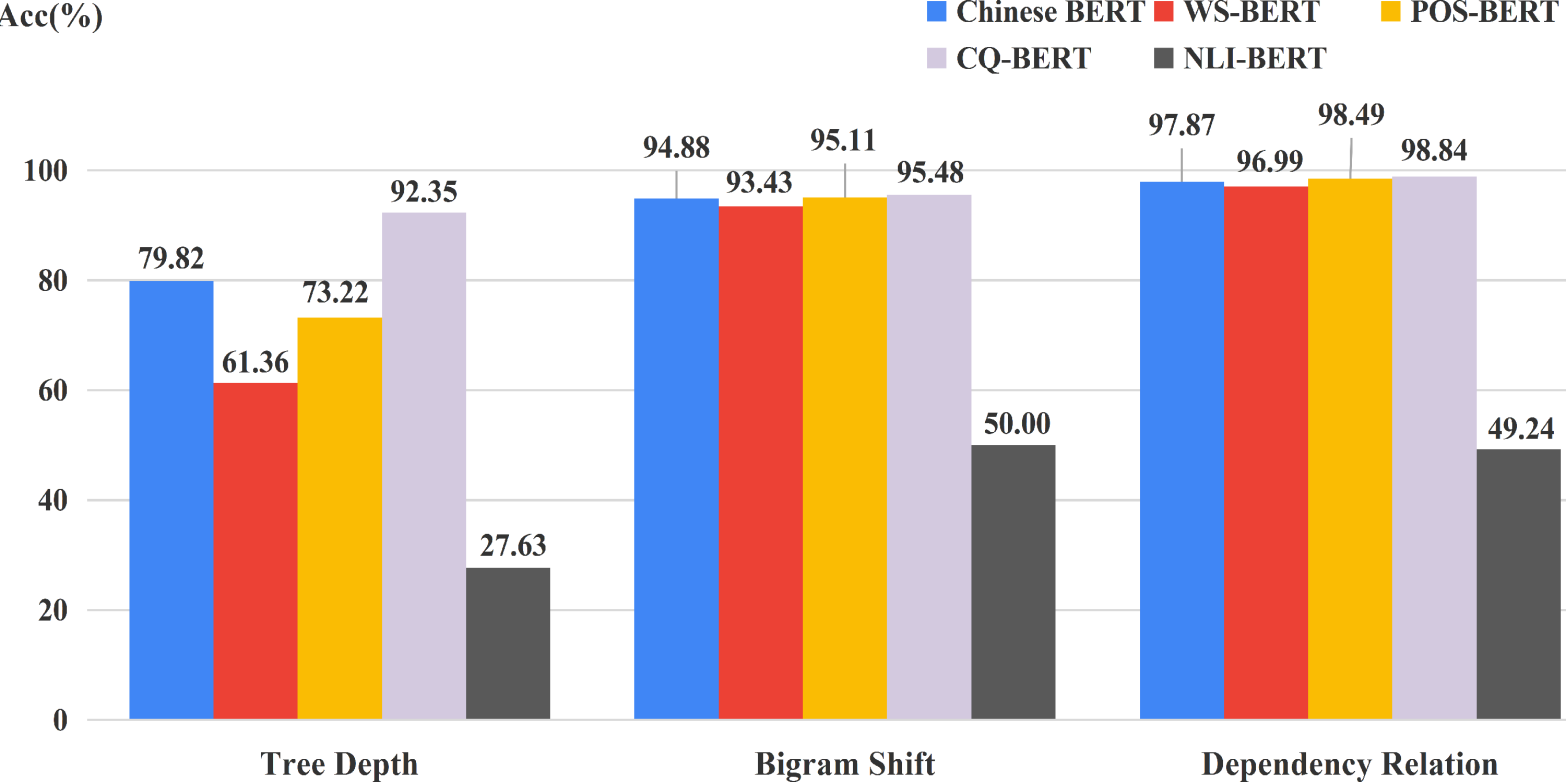
Accuracy on three syntactic tasks for fine-tuned models and Chinese BERT.

Figure 10 displays the best performance among all 12 layers of each BERT on three syntactic tasks. NLI-BERT still performed very poor. Chinese BERT still outperformed WS-BERT on all tasks, which indicates that the syntactic knowledge in hidden states of WS-BERT could be forgotten to some extent. Very interestingly, both POS-BERT and CQ-BERT showed improvement on BShift and DepRel. However, only CQ-BERT surpassed Chinese BERT on Tree Depth. The reason could be that POS-BERT might capture some local information about the relations between words. Hence, POS-BERT is very suitable to BShift and DepRel tasks. CQ-BERT can learn the organization structure of the whole sentence better, Therefore, this model can acquire more obvious progress on the TreeDepth task.

## Conclusion

We explored the competence of Chinese BERT in encoding syntactic knowledge across two aspects: attention heads and hidden states. We observed that certain attention heads learned specific dependency relations and syntactic phenomena. By combining attention heads, we succeeded in parsing the sentences. Hidden states also reflected some competence in encoding syntactic knowledge. When Chinese BERT was fine-tuned into different downstream tasks, we found some changes of different models in preserving language structure. POS tagging reinforced syntactic information in Chinese BERT to some extent, while NLI enabled Chinese BERT to lose knowledge in learning sentence structure.

Those findings above can guide the design of model distillation algorithms in term of those heads encoding syntactic knowledge. Furthermore, we can be aware that whether syntactic knowledge is of importance when finishing a specific NLP downstream task. Meanwhile, some specific syntactic information can be introduced more precisely to improve the task performance according to our findings.

## Additional Information and Declarations

### Data Availability

The following information was supplied regarding data availability: The code is available at GitHub and Zenodo:
https://github.com/JianyuZheng/Syntactic-Knowledge-in-Chinese-BERT. JianyuZheng. (2023). JianyuZheng/Syntactic-Knowledge-in-Chinese-BERT: Python (Python). Zenodo. https://doi.org/10.5281/zenodo.7878343. The data is available at the LINDAT/CLARIAH-CZ Repository, Zenodo, and the Linguistic Data Consortium: Zeman, Daniel; et al., 2022, Universal Dependencies 2.11, LINDAT/CLARIAH-CZ digital library at the Institute of Formal and Applied Linguistics (ÚFAL), Faculty of Mathematics and Physics, Charles University, http://hdl.handle.net/11234/1-4923. Jianyu Zheng. (2023). Three syntactic probing tasks for hidden states [Data set]. Zenodo. https://doi.org/10.5281/zenodo.7878395. Che, Wanxiang, Zhenghua Li, and Ting Liu. Chinese Dependency Treebank 1.0 LDC2012T05. Web Download. Philadelphia: Linguistic Data Consortium, 2012. https://catalog.ldc.upenn.edu/LDC2012T05.
